# Cancer-related fatigue trajectories up to 5 years after curative treatment for oesophageal cancer

**DOI:** 10.1038/s41416-023-02551-0

**Published:** 2023-12-22

**Authors:** Zhao Cheng, Asif Johar, Magnus Nilsson, Anna Schandl, Pernilla Lagergren

**Affiliations:** 1 Surgical Care Science, Department of Molecular Medicine and Surgery, Karolinska Institutet, Karolinska University Hospital, Stockholm, Sweden; 2 Division of Surgery, Department of Clinical Science, Intervention and Technology (CLINTEC), Karolinska Institutet, Stockholm, Sweden; 3 Department of Upper Abdominal Diseases, Karolinska University Hospital, Stockholm, Sweden; 4 Department of Surgery and Cancer, Imperial College London, London, UK

**Keywords:** Quality of life, Oesophageal cancer, Risk factors, Fatigue

## Abstract

**Background:**

Whether cancer-related fatigue develops differently after curative-intended oesophageal cancer treatment and the related modifiable factors are unclear.

**Methods:**

This population-based and longitudinal cohort included 409 oesophageal cancer patients who underwent curative oesophagectomy in 2013–2020 in Sweden. The main outcome was cancer-related fatigue trajectories with measurements at 1, 1.5, 2, 2.5, 3, 4 and 5 years postoperatively by validated EORTC QLQ-FA12 questionnaire, and analysed using growth mixture models. Weighted logistic regressions provided odds ratios (OR) with 95% confidence intervals (95% CI) for underlying sociodemographic, clinical, and patient-reported outcome factors in relation to the identified trajectories.

**Results:**

Two distinct overall cancer-related fatigue trajectories were identified: low level of persistent fatigue and high level of increasing fatigue, with 64% and 36% of patients, respectively. The odds of having high level of fatigue trajectory were increased by Charlson comorbidity index (≥ 2 versus 0: OR = 2.52, 95% CI 1.07–5.94), pathological tumour Stage (III–IV versus 0-I: OR = 2.52, 95% CI 1.33–4.77), anxiety (OR = 7.58, 95% CI 2.20–26.17), depression (OR = 15.90, 95% CI 4.44–56.93) and pain (continuous score: OR = 1.02, 95% CI 1.01–1.04).

**Conclusions:**

Long-term trajectories with high level of increasing cancer-related fatigue and the associated modifiable factors were identified after oesophageal cancer treatment. The results may facilitate early identification and targeted intervention for such high-risk patients.

## Introduction

Oesophageal cancer is one of the leading causes of cancer incidence and mortality globally [1]. Surgery is the mainstay of curatively intended treatment, usually combined with neoadjuvant therapy for locally advanced oesophageal cancer [2]. The treatment is challenging and extensive, often followed by diminished health-related quality of life (HRQL) [2]. The 5-year overall survival for oesophageal cancer patients is <20% worldwide [3–5], and the 5-year survival reaches 30–50% after the curative-intent oesophagectomy [6–8]. With the prolonged survival, the accompanying long-term survivorship is of increasing importance to clarify.

Cancer-related fatigue refers to the distressing, subjective feeling of tiredness related to cancer or cancer treatment, that is not proportional to recent activity and interferes with daily-life functioning. Cancer-related fatigue is a multidimensional symptom, comprising physical, emotional, and cognitive components [9, 10]. It is one of the most common, severe and persistent HRQL-related symptoms in oesophageal cancer patients [11–13]. The aetiology of cancer-related fatigue is rather vague and evidence suggests a multifactorial process involving demographic, physiological, medical, psychosocial, and behavioural factors [14, 15]. Longitudinal research on cancer-related fatigue in oesophageal cancer survivors is scarce and classic approaches focusing on the average population level may conceal the heterogeneity in cancer-related fatigue development over time.

This study aims to explore the potentially distinct trajectories of cancer-related fatigue among oesophageal cancer survivors until five years after oesophagectomy and the potential factors associated with the long-term trajectories. Such evidence could assist healthcare professionals, patients, and family caregivers with a better understanding and preparation for cancer-related fatigue, contribute to the early identification of high-risk groups, and facilitate prompt interventions and targeted follow-up.

## Methods

### Study design

This is an ongoing Swedish nationwide and prospective cohort study entitled “Oesophageal Surgery on Cancer patients-Adaptation and Recovery (OSCAR) study” [11, 16]. The cohort enrolled all patients with oesophageal cancer who underwent oesophagectomy between January 1, 2013, and June 30, 2020, in Sweden. OSCAR follows up the patients regularly from 1 to 12 years after surgery. For the purpose of this study, all available data until June 30, 2022, i.e., up to 5 years after oesophagectomy was used, despite some patients having more than 5-year follow-up. The project was approved by the Regional Ethical Review Board in Stockholm (2013/844-31/1). Informed consent forms were obtained from all participants.

### Data source and collection

Patients were identified via pathology departments at all 8 hospitals performing surgery for oesophageal cancer in Sweden, and those who were alive 1 year after oesophagectomy were included. Cancer-related fatigue was reported by patients at 1, 1.5, 2, 2.5, 3, 4 and 5 years postoperatively. At 1 and 5 years, a research nurse visited the patients and conducted personal interviews using computer-based questionnaires, and patients responded to mailed questionnaires during other follow-ups. During the 1-year follow-up, patient-reported information was collected on anxiety, depression, pain, insomnia, physical activity, height and average weight as an adult. Vital status was retrieved from the National Register of the Total Population with deterministic ascertainment by patient’s unique identification number. Age, sex and education information were obtained from the longitudinal integrated database for health insurance and labour market studies (LISA) using patient’s unique identification number. To estimate the proxy baseline fatigue level (before diagnosis) for the oesophageal cancer patients, 6969 random samples from the Swedish population (reference population) were selected, 4910 (70.5%) of whom participated and 4867 (99.1%) responded to the same questionnaire (EORTC QLQ-C30 fatigue subscale) as patients [17]. Each patient in OSCAR was individually matched to more than 50 persons from the reference population by age at surgery (5-year time window), sex, education level (< 9, 9–12 or >12 years of formal education), and comorbidities (cardiovascular, respiratory, diabetes and others). The proxy baseline fatigue level for each oesophageal cancer patient was calculated as the mean score of cancer-related fatigue from the matched individuals. Clinical data, including comorbidity, tumour histology, treatment, pathological tumour stage, postoperative complications and weight at the time of operation were collected by a review of medical records (histopathology reports, operation charts and discharge notes) according to a predefined protocol.

### Outcomes

Cancer-related fatigue was the study outcome, measured by two validated questionnaires developed by the European Organization for Research and Treatment of Cancer (EORTC): Fatigue subscale of the EORTC Quality of Life Core Questionnaire 30 (EORTC QLQ-C30) and the EORTC QLQ-Fatigue 12 (EORTC QLQ-FA12) [18, 19]. The official Swedish translation versions of the questionnaires, with consistent translation and cultural equivalence of the measures, were used in this study [20]. The 30-item EORTC QLQ-C30 questionnaire evaluates HRQL in cancer patients, including a three-item subscale measuring cancer-related fatigue. EORTC QLQ-FA12 is a 12-item multidimensional questionnaire, which is supplemented with the EORTC QLQ-C30 in specific detail. QLQ-FA12 incorporates three multi-item scales measuring physical, emotional, and cognitive aspects of cancer-related fatigue, and two single items to assess interference with daily life (whether the tiredness interferes with patients’ daily activities) and social sequelae (whether the tiredness is not understood by the people who are close to patients). The primary outcomes were cancer-related fatigue trajectories (categorical variable) based on QLQ-C30 fatigue and QLQ-FA12 overall fatigue. The secondary outcomes were trajectories (categorical variable) based on the 3 symptom scales and 2 single items in QLQ-FA12. Fatigue questionnaire responses were transformed into 0-100 scores. Higher scores indicate more cancer-related fatigue. Missing data were handled in line with the EORTC scoring manual [21].

### Underlying factors

Nine predefined sociodemographic and clinical factors that were considered to potentially influence the cancer-related fatigue trajectory were included: Age at surgery (continuous variable), sex (male or female), education level (< 9, 9–12 or >12 years of formal education), proxy baseline QLQ-C30 fatigue score (continuous variable), comorbidity (Charlson comorbidity index 0, 1 or ≥2, excluding oesophageal cancer) [22], tumour histology (squamous cell carcinoma or adenocarcinoma), neoadjuvant chemo(radio)therapy (no or yes), pathological tumour Stage (0-I, II or III–IV), and 30-day postoperative complications (Clavien–Dindo classifications 0–I, II–IIIa or IIIb–IV) [23].

Six additional patient-reported outcome factors were also included: anxiety (Hospital Anxiety and Depression Scale [HADS] anxiety score ≥8 was regarded as clinically relevant anxiety symptom: no or yes) [24], depression (HADS depression score ≥8 was regarded as clinically relevant depression symptom: no or yes), pain score (EORTC QLQ-C30, continuous variable), insomnia score (EORTC QLQ-C30, continuous variable), preoperative body mass index (BMI) adjusted weight loss grading system (categorised by BMI at operation and weight loss between average weight as an adult and at the time of operation: 0, 1, 2, 3 or 4) [25], and physical activity one year after oesophagectomy (International Physical Activity Questionnaire [IPAQ]: low, moderate, and high level) [26]. In particular, pain and insomnia were analysed as a continuous score, so their effect size was calculated by each score increase and was expected to be small (a statistically significant small OR might still indicate meaningful associations). These factors were selected based on existing literature [10, 12, 27–34] and availability in the study cohort.

### Statistical analysis

Growth mixture models were used to identify separable unobserved (latent) trajectories of cancer-related fatigue, a latent-class analysing method that could identify subgroups following similar longitudinal courses of the outcome within a heterogeneous population [35–38]. First, the model was fitted with a single linear trajectory. Trajectory numbers were then gradually increased until the model reached the best-identified performance. Up to four trajectories, different latent or residual variances, and trajectory shapes (linear or quadratic) were fitted and compared for each outcome. Model performance was assessed by a combination of the following criteria: Akaike Information Criterion (AIC), Bayesian Information Criterion (BIC), sample-size adjusted BIC, entropy, Vuong–Lo–Mendell–Rubin test (VLMR), adjusted Lo–Mendell–Rubin test (aLMR), and model interpretability [39]. BIC, sample-size adjusted BIC, and AIC compare the log-likelihood of nested models, and smaller values imply better model fit. Entropy represents the trajectory classification uncertainty, where higher values indicate better accuracy. VLMR and aLMR compare the K-trajectory with the (K-1)-trajectory models and a significant *P* value (≤0.05) favours the fit of the K-trajectory model. Models with reasonable interpretation and better statistical index were selected. Models with a trajectory size of less than 15% of the patients were rejected. The posterior probabilities of trajectory membership for each patient were estimated by the selected models, and patients were assigned to the trajectory with the highest posterior probabilities, i.e., the most likely trajectory for the patient. The model-estimated mean and sample mean for each trajectory were output from the growth mixture models. The sample mean was calculated as the mean of fatigue scores with the posterior probabilities in each trajectory as weights. Patients with at least one cancer-related fatigue measurement were included, and all available data were used in the growth mixture models, assuming that data are missing at random.

Weighted logistic regression models calculated odds ratios (OR) with 95% confidence intervals (CI) to estimate associations between underlying factors and cancer-related fatigue trajectories. The reference outcome trajectory was the trajectory with the lowest level of cancer-related fatigue. The selected factors were included in multivariable models and assumed to be confounders for each other. Factors were included step by step: sociodemographic and clinical factors were first included, and then patient-reported outcome factors (anxiety, depression, pain and insomnia) were also added to the models. Due to major missing in the factors preoperative body mass index (BMI) adjusted weight loss grading system and IPAQ physical activity, these two were added to the model in the last step. The weights used were the posterior probabilities of the assigned trajectories from growth mixture models. Statistical significance was tested at two-sided 5% levels. Sensitivity analyses were conducted regarding QLQ-C30 fatigue and QLQ-FA12 overall fatigue, excluding patients who had only one fatigue measurement. Growth mixture models were analysed by MPlus version 8.7 software (Los Angeles, California: Muthén & Muthén), and all other analyses were done using SAS version 9.4 software (Cary, North Carolina: SAS Institute Inc.).

## Results

### Patients

**Fig. 1:**
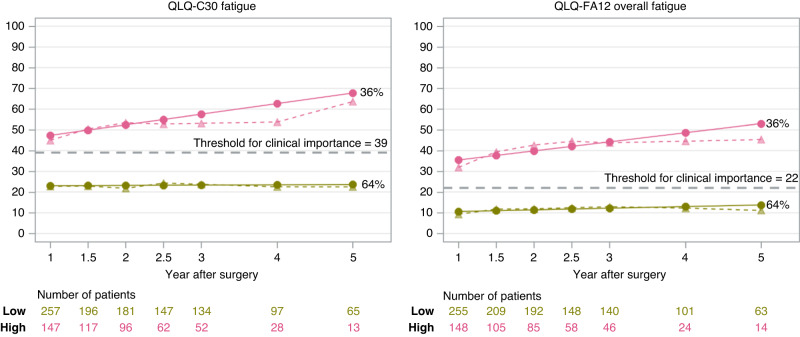
Cancer-related fatigue trajectories after surgery for oesophageal cancer. (1) Solid lines represent estimated means. The dotted lines represent sample means. (2) The percentage after each trajectory is the final patient proportion for the trajectory category based on the most likely trajectory membership. (3) Grey dashed lines represent thresholds for clinical importance (Giesinger et al. [40]; Friedrich et al. [41]).

**Fig. 2:**
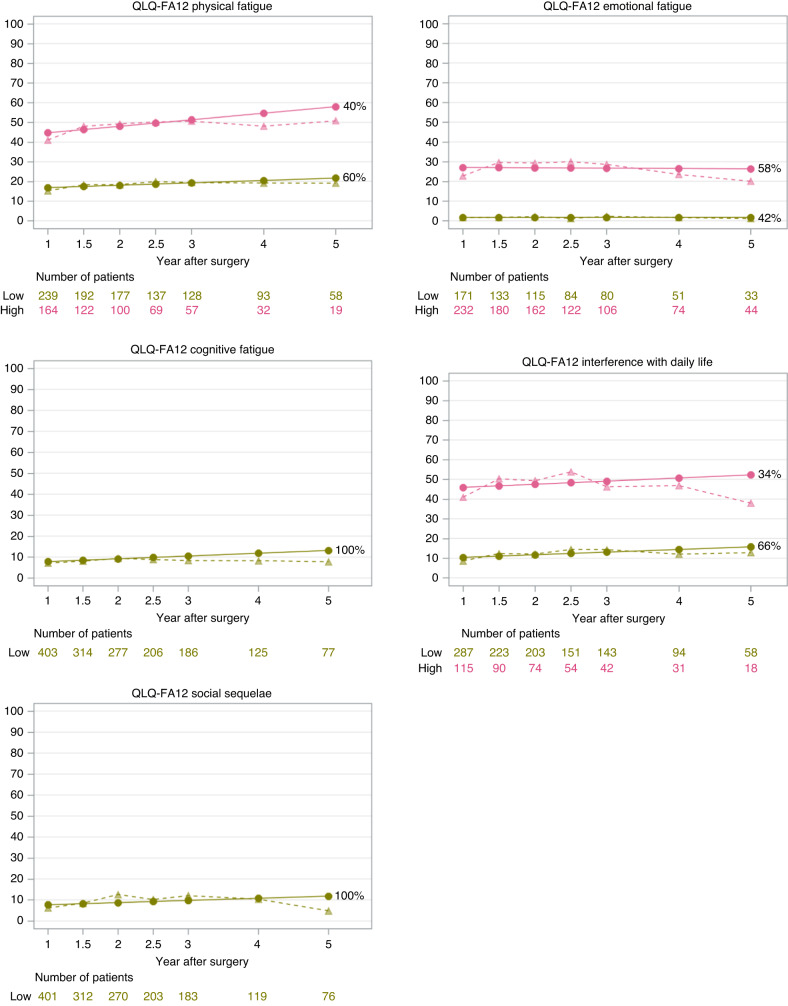
Cancer-related fatigue sub-dimension trajectories after surgery for oesophageal cancer. (1) Solid lines represent estimated means. The dotted lines represent sample means. (2) The percentage after each trajectory is the final patient proportion for the trajectory category based on the most likely trajectory membership.

Among the 1013 oesophageal cancer patients who underwent oesophagectomy between January 1, 2013, and June 30, 2020, in Sweden, 242 (23.9%) died within one year after surgery, and 154 (15.2%) were unreachable, leaving 617 eligible patients for study inclusion. Of these, 143 (23.2%) declined to consent, 30 (4.9%) were too sick to participate, 26 (4.2%) had tumour recurrence, and therefore 418 (67.7%) patients participated in the study. Cancer-related fatigue measurements were missing in 9 of the participating patients, and the final study cohort incorporated 409 patients who responded to at least one of the postoperative QLQ-C30 fatigue measurements. At 1 year after oesophagectomy, 404 patients completed the questionnaire, followed by 314 (77.7%), 277 (88.2%), 209 (75.5%), 186 (89.0%), 125 (67.2%), 78 (62.4%) at 1.5, 2, 2.5, 3, 4 and 5 years. The respective attrition numbers for each fatigue measurement are also shown in Figs. 1 and  2.

The mean age of the participants was 67.2 years, and the majority were men (91.7%), had a tumour of adenocarcinoma histology (86.1%), and underwent neoadjuvant Chemo(radio)therapy (80.4%) (Supplementary Table S1).

### Cancer-related fatigue trajectories

Two similar distinct trajectories were identified for the primary outcomes of QLQ-C30 fatigue and QLQ-FA12 overall fatigue: 64% of the patients experienced a persistently low level of fatigue, while 36% experienced a higher level of fatigue which seemed to increase with time (Fig. 1). The trajectories of high-level fatigue regarding QLQ-C30 and QLQ-FA12 overall measurements were above the threshold for clinical importance (Fig. 1) [40, 41]. As for the specific aspects of cancer-related fatigue (Fig. 2), two trajectories were identified for physical fatigue: persistently low levels of symptoms and a higher level of symptoms increase with time. Two trajectories were identified for emotional fatigue and interference with daily life: low and high level of symptoms that were persistent over time. Only one trajectory with a low level of persistent symptoms was identified for cognitive fatigue and social sequelae. Fit parameters for model selection are provided in Supplementary Table S2.

Patients’ characteristics within each cancer-related fatigue trajectory are presented in Table 1. Patients with more comorbidities, advanced pathological tumour stage, higher Clavien–Dindo classification, anxiety, depression, more pain and insomnia symptoms were overrepresented in the high level of cancer-related fatigue trajectory.

**Table 1:** Characteristics of 409 patients in the final study cohort by cancer-related fatigue trajectories.

	QLQ-C30 fatigue trajectory^a^			QLQ-FA12 overall fatigue trajectory^b^		
	Low^c^	High^c^		Low^c^	High^c^	
	*N* = 261, number (%)	*N* = 148, number (%)	*p*	*N* = 260, number (%)	*N* = 148, number (%)	*P*
Age						
Mean (standard deviation)	67.9 (7.4)	66.0 (9.6)	0.037	68.0 (7.3)	66.0 (9.7)	0.029
Sex						
Female	20 (7.7)	14 (9.5)	0.527	20 (7.7)	14 (9.5)	0.535
Male	241 (92.3)	134 (90.5)		240 (92.3)	134 (90.5)	
Education level (years)						
<9	63 (24.1)	37 (25.0)	0.721	68 (26.2)	31 (20.9)	0.612
9–12	123 (47.1)	67 (45.3)		116 (44.6)	74 (50.0)	
>12	73 (28.0)	44 (29.7)		75 (28.8)	42 (28.4)	
Missing	2 (0.8)	0 (0.0)		1 (0.4)	1 (0.7)	
Proxy baseline QLQ-C30 fatigue^a^						
Mean (standard deviation)	14.9 (7.1)	15.6 (6.8)	0.320	15.0 (7.1)	15.6 (6.8)	0.366
Charlson comorbidity index						
0	111 (42.5)	55 (37.2)	0.553	114 (43.8)	52 (35.1)	0.102
1	85 (32.6)	49 (33.1)		85 (32.7)	49 (33.1)	
≥2	56 (21.5)	40 (27.0)		52 (20.0)	44 (29.7)	
Missing	9 (3.4)	4 (2.7)		9 (3.5)	3 (2.0)	
Tumour histology						
Squamous cell carcinoma	33 (12.6)	23 (15.5)	0.289	36 (13.8)	20 (13.5)	0.747
Adenocarcinoma	228 (87.4)	124 (83.8)		223 (85.8)	128 (86.5)	
Missing	0 (0.0)	1 (0.7)		1 (0.4)	0 (0.0)	
Chemo(radio)therapy						
No	48 (18.4)	31 (20.9)	0.625	49 (18.8)	30 (20.3)	0.712
Yes	212 (81.2)	117 (79.1)		210 (80.8)	118 (79.7)	
Missing	1 (0.4)	0 (0.0)		1 (0.4)	0 (0.0)	
Pathological tumour stage						
0-I	98 (37.5)	39 (26.4)	0.055	99 (38.1)	38 (25.7)	0.002
II	80 (30.7)	45 (30.4)		85 (32.7)	40 (27.0)	
III–IV	81 (31.0)	61 (41.2)		73 (28.1)	69 (46.6)	
Missing	2 (0.8)	3 (2.0)		3 (1.2)	1 (0.7)	
Clavien–Dindo classification						
0–I	101 (38.7)	47 (31.8)	0.255	100 (38.5)	48 (32.4)	0.317
II–IIIa	93 (35.6)	54 (36.5)		89 (34.2)	58 (39.2)	
IIIb–IV	58 (22.2)	44 (29.7)		62 (23.8)	40 (27.0)	
Missing	9 (3.4)	3 (2.0)		9 (3.5)	2 (1.4)	
HADS anxiety						
No	242 (92.7)	117 (79.1)	<0.001	249 (95.8)	110 (74.3)	<0.001
Yes	15 (5.7)	29 (19.6)		6 (2.3)	38 (25.7)	
Missing	4 (1.5)	2 (1.4)		5 (1.9)	0 (0.0)	
HADS depression						
No	245 (93.9)	110 (74.3)	<0.001	249 (95.8)	106 (71.6)	<0.001
Yes	12 (4.6)	36 (24.3)		6 (2.3)	42 (28.4)	
Missing	4 (1.5)	2 (1.4)		5 (1.9)	0 (0.0)	
QLQ-C30 pain^a^						
Mean (standard deviation)	13.9 (20.3)	29.6 (29.1)	<0.001	13.2 (19.5)	30.5 (29.3)	<0.001
Number of missing	4	1		5	0	
QLQ-C30 insomnia^a^						
Mean (standard deviation)	18.7 (26.8)	32.7 (35.6)	<0.001	19.2 (27.9)	31.1 (34.0)	<0.001
Number of missing	4	1		5	0	
IPAQ physical activity						
Low	65 (24.9)	42 (28.4)	0.157	61 (23.5)	46 (31.1)	0.237
Moderate	86 (33.0)	37 (25.0)		83 (31.9)	40 (27.0)	
High	76 (29.1)	40 (27.0)		79 (30.4)	37 (25.0)	
Missing	34 (13.0)	29 (19.6)		37 (14.2)	25 (16.9)	
Preoperative BMI adjusted weight loss grading system						
0	91 (34.9)	51 (34.5)	0.621	89 (34.2)	53 (35.8)	0.474
1	28 (10.7)	20 (13.5)		28 (10.8)	20 (13.5)	
2	38 (14.6)	29 (19.6)		38 (14.6)	29 (19.6)	
3	51 (19.5)	23 (15.5)		50 (19.2)	24 (16.2)	
4	24 (9.2)	11 (7.4)		25 (9.6)	10 (6.8)	
Missing	29 (11.1)	14 (9.5)		30 (11.5)	12 (8.1)	

*HADS* Hospital Anxiety and Depression Scale, *IPAQ* International Physical Activity Questionnaire, *BMI* body mass index.

^a^QLQ-C30: Quality of Life Core Questionnaire.

^b^QLQ-FA12: Quality of Life Fatigue Questionnaire.

^c^“Low” and “high” refer to the identified trajectories presented in Fig. 1.

### Factors associated with cancer-related fatigue trajectories

Older age was associated with having a low level of QLQ-C30 fatigue (OR = 0.96, 95% CI 0.94–0.99), QLQ-FA12 overall (OR 0.96, 95% CI 0.93–0.99), physical (OR = 0.97, 95% CI 0.94–1.00), and emotional (OR = 0.97, 95% CI 0.94–0.99) fatigue trajectory (Table 2). Having more comorbidities was associated with a higher level of QLQ-FA12 overall fatigue (Charlson comorbidity index ≥2 versus 0, those with index, OR = 2.73, 95% CI 1.29–5.78). Pathological tumour Stage III–IV was associated with a higher level of QLQ-C30 fatigue (OR = 2.25, 95% CI 1.28–3.97), QLQ-FA12 overall (OR = 2.33, 95% CI 1.36–4.01) and physical (OR = 1.83, 95% CI 1.08–3.12) fatigue trajectory, compared to Stages 0–I. Patients with more early postoperative complications were more likely to have a high level of emotional fatigue trajectory (Clavien–Dindo classification II–IIIa versus 0–I: OR = 1.78, 95% CI 1.09–2.91) (Table 2).

**Table 2:** Odds ratios (95% confidence intervals) between sociodemographic, clinical factors, and cancer-related fatigue^a^ trajectories after surgery for oesophageal cancer.

	QLQ-C30 fatigue^b^	QLQ-FA12 overall fatigue^c^	QLQ-FA12 physical fatigue^c^	QLQ-FA12 emotional fatigue^c^	QLQ-FA12 interference with daily life^c^
Reference trajectory	Low	Low	Low	Low	Low
Outcome trajectory	High	High	High	High	High
Age					
Continuous	**0.96 (0.94–0.99)**	**0.96 (0.93–0.99)**	**0.97 (0.94–1.00)**	**0.97 (0.94–0.99)**	0.97 (0.95–1.00)
Sex					
Female	1.00 (Reference)	1.00 (Reference)	1.00 (Reference)	1.00 (Reference)	1.00 (Reference)
Male	0.89 (0.37–2.10)	0.88 (0.38–2.06)	0.60 (0.27–1.35)	0.67 (0.29–1.52)	0.52 (0.23–1.18)
Education level (years)					
<9	1.00 (Reference)	1.00 (Reference)	1.00 (Reference)	1.00 (Reference)	1.00 (Reference)
9–12	0.99 (0.52–1.89)	1.27 (0.67–2.40)	0.93 (0.50–1.73)	1.34 (0.74–2.43)	1.11 (0.58–2.14)
>12	0.90 (0.50–1.61)	1.27 (0.71–2.26)	1.03 (0.59–1.80)	1.18 (0.70–2.00)	1.02 (0.56–1.86)
Proxy baseline QLQ-C30 fatigue^b^					
Continuous	1.00 (0.95–1.04)	0.98 (0.94–1.02)	1.00 (0.95–1.04)	0.99 (0.95–1.04)	1.00 (0.95–1.04)
Charlson comorbidity index					
0	1.00 (Reference)	1.00 (Reference)	1.00 (Reference)	1.00 (Reference)	1.00 (Reference)
1	1.35 (0.76–2.39)	1.63 (0.93–2.86)	1.22 (0.71–2.11)	1.43 (0.85–2.40)	1.54 (0.85–2.78)
≥2	1.55 (0.73–3.31)	**2.73 (1.29–5.78)**	1.87 (0.90–3.90)	1.54 (0.76–3.15)	**2.56 (1.19–5.50)**
Tumour histology					
Squamous cell carcinoma	1.00 (Reference)	1.00 (Reference)	1.00 (Reference)	1.00 (Reference)	1.00 (Reference)
Adenocarcinoma	0.71 (0.36–1.39)	0.90 (0.45–1.80)	0.86 (0.44–1.68)	0.84 (0.44–1.62)	0.70 (0.35–1.41)
Chemo(radio)therapy					
No	1.00 (Reference)	1.00 (Reference)	1.00 (Reference)	1.00 (Reference)	
Yes	0.83 (0.46–1.52)	0.91 (0.51–1.62)	0.90 (0.51–1.60)	1.03 (0.60–1.79)	1.10 (0.59–2.04)
Pathological tumour stage					
0–I	1.00 (Reference)	1.00 (Reference)	1.00 (Reference)	1.00 (Reference)	1.00 (Reference)
II	1.59 (0.89–2.85)	1.23 (0.69–2.17)	1.19 (0.69–2.07)	1.01 (0.60–1.70)	1.27 (0.70–2.30)
III–IV	**2.25 (1.28–3.97)**	**2.33 (1.36–4.01)**	**1.83 (1.08–3.12)**	1.12 (0.67–1.87)	1.59 (0.89–2.82)
Clavien–Dindo classification					
0–I	1.00 (Reference)	1.00 (Reference)	1.00 (Reference)	1.00 (Reference)	1.00 (Reference)
II–IIIa	1.26 (0.74–2.16)	1.35 (0.80–2.28)	1.05 (0.63–1.75)	**1.78 (1.09–2.91)**	1.24 (0.72–2.14)
IIIb–IV	1.53 (0.85–2.77)	1.31 (0.73–2.33)	1.31 (0.75–2.31)	1.56 (0.91–2.68)	1.06 (0.57–1.95)

^a^Cognitive fatigue is not included in the analysis since only one trajectory was identified.

^b^QLQ-C30: Quality of Life Core Questionnaire.

^c^QLQ-FA12: Quality of Life Fatigue Questionnaire.

Bold results are statistically significant.

When further including patient-reported outcome factors in the multivariable models (Table 3), the effects of the Charlson comorbidity index, pathological tumour stage, and Clavien–Dindo classification remained as displayed in Table 2, while the effect of age disappeared. Anxiety was associated with 7 times the odds of having a high level of QLQ-FA12 overall fatigue trajectory (OR = 7.58, 95% CI 2.20–26.17). Depression was associated with about 15-fold increased odds of a high level of QLQ-FA12 overall fatigue trajectory (OR = 15.90, 95% CI 4.44–56.93). Patients with more pain symptoms also showed increased odds of higher-level trajectories for all cancer-related fatigue measurements. Patients with more insomnia symptoms had increased odds of having higher-level trajectories for QLQ-C30 fatigue (OR = 1.01, 95% CI 1.00–1.02) and QLQ-FA12 emotional fatigue (OR = 1.01, 95% CI 1.00–1.02). Preoperative BMI adjusted weight loss grading system and physical activity level 1 year after the surgery did not seem to be associated with cancer-related trajectories (Supplementary Table S3).

**Table 3:** Odds ratios (95% confidence intervals) between sociodemographic, clinical and patient-reported outcome factors in relation to cancer-related fatigue^a^ trajectories after surgery for oesophageal cancer.

	QLQ-C30 fatigue^b^	QLQ-FA12 overall fatigue^c^	QLQ-FA12 physical fatigue^c^	QLQ-FA12 emotional fatigue^c^	QLQ-FA12 interference with daily life^c^
Reference trajectory	Low	Low	Low	Low	Low
Outcome trajectory	High	High	High	High	High
Age					
Continuous	1.00 (0.97–1.03)	1.00 (0.97–1.04)	1.00 (0.97–1.04)	1.00 (0.97–1.04)	1.01 (0.98–1.05)
Sex					
Female	1.00 (Reference)	1.00 (Reference)	1.00 (Reference)	1.00 (Reference)	1.00 (Reference)
Male	1.07 (0.41–2.79)	1.54 (0.53–4.53)	0.74 (0.29–1.84)	0.89 (0.34–2.37)	0.67 (0.26–1.68)
Education level (years)					
<9	1.00 (Reference)	1.00 (Reference)	1.00 (Reference)	1.00 (Reference)	1.00 (Reference)
9–12	0.95 (0.47–1.92)	1.25 (0.59–2.66)	0.86 (0.43–1.71)	1.50 (0.77–2.93)	1.09 (0.52–2.29)
>12	0.81 (0.43–1.53)	1.24 (0.62–2.45)	0.95 (0.51–1.74)	1.12 (0.62–2.03)	0.99 (0.51–1.95)
Proxy baseline QLQ-C30 fatigue^b^					
Continuous	1.00 (0.96–1.05)	0.98 (0.93–1.03)	1.00 (0.96–1.05)	1.00 (0.95–1.05)	1.00 (0.96–1.05)
Charlson comorbidity index					
0	1.00 (Reference)	1.00 (Reference)	1.00 (Reference)	1.00 (Reference)	1.00 (Reference)
1	0.97 (0.51–1.84)	0.97 (0.49–1.92)	0.80 (0.43–1.49)	1.09 (0.61–1.96)	1.03 (0.52–2.02)
≥2	1.39 (0.61–3.14)	**2.52 (1.07–5.94)**	1.63 (0.74–3.60)	1.36 (0.62–2.98)	2.32 (0.99–5.39)
Tumour histology					
Squamous cell carcinoma	1.00 (Reference)	1.00 (Reference)	1.00 (Reference)	1.00 (Reference)	1.00 (Reference)
Adenocarcinoma	0.97 (0.45–2.09)	1.41 (0.61–3.27)	1.22 (0.57–2.62)	1.14 (0.54–2.42)	0.92 (0.42–1.99)
Chemo(radio)therapy					
No	1.00 (Reference)	1.00 (Reference)	1.00 (Reference)	1.00 (Reference)	1.00 (Reference)
Yes	1.04 (0.54–2.02)	1.43 (0.70–2.92)	1.16 (0.61–2.22)	1.42 (0.76–2.64)	1.61 (0.78–3.31)
Pathological tumour stage					
0–I	1.00 (Reference)	1.00 (Reference)	1.00 (Reference)	1.00 (Reference)	1.00 (Reference)
II	1.61 (0.85–3.07)	1.24 (0.63–2.45)	1.20 (0.65–2.22)	0.96 (0.54–1.72)	1.30 (0.66–2.54)
III–IV	**2.16 (1.16–4.01)**	**2.52 (1.33–4.77)**	1.74 (0.97–3.14)	0.93 (0.52–1.64)	1.52 (0.80–2.91)
Clavien–Dindo classification					
0–I	1.00 (Reference)	1.00 (Reference)	1.00 (Reference)	1.00 (Reference)	1.00 (Reference)
II–IIIa	1.45 (0.80–2.61)	1.56 (0.84–2.90)	1.12 (0.64–1.97)	**2.10 (1.21–3.64)**	1.45 (0.79–2.67)
IIIb–IV	1.90 (0.99–3.67)	1.64 (0.82–3.27)	1.53 (0.82–2.85)	**1.95 (1.06–3.57)**	1.28 (0.64–2.55)
HADS anxiety					
No	1.00 (Reference)	1.00 (Reference)	1.00 (Reference)	1.00 (Reference)	1.00 (Reference)
Yes	1.58 (0.60–4.14)	**7.58 (2.20–26.17)**	2.43 (0.88–6.69)	4.80 (0.99–23.23)	**3.93 (1.55–10.01)**
HADS depression					
No	1.00 (Reference)	1.00 (Reference)	1.00 (Reference)	1.00 (Reference)	1.00 (Reference)
Yes	**5.22 (1.98–13.76)**	**15.90 (4.44–56.93)**	**8.41 (2.78–25.41)**	**10.60 (2.09–53.77)**	**5.95 (2.45–14.46)**
QLQ-C30 pain^b^					
Continuous	**1.02 (1.01–1.03)**	**1.02 (1.01–1.04)**	**1.02 (1.01–1.03)**	**1.03 (1.01–1.04)**	**1.02 (1.00–1.03)**
QLQ-C30 insomnia^b^					
Continuous	**1.01 (1.00–1.02)**	1.01 (1.00–1.02)	1.01 (1.00–1.01)	**1.01 (1.00–1.02)**	1.01 (1.00–1.02)

*HADS* Hospital Anxiety and Depression Scale.

^a^Cognitive fatigue is not included in the analysis since only one trajectory was identified.

^b^QLQ-C30: Quality of Life Core Questionnaire.

^c^QLQ-FA12: Quality of Life Fatigue Questionnaire.

Bold results are statistically significant.

### Sensitivity analyses

There were 71 (17%) out of the 409 patients who had a single measurement for fatigue. After excluding them, the identified trajectories (Supplementary Fig. S1) and the associations with the potential factors (Supplementary Table S4) remained similar as the main results.

## Discussion

Two distinct long-term trajectories of cancer-related fatigue were identified among oesophageal cancer survivors after oesophagectomy, and different longitudinal patterns were presented by specific fatigue dimensions. Clinical factors, including more comorbidities, advanced pathological tumour stage, and postoperative complications, as well as modifiable patient-reported outcome factors one year after surgery, especially anxiety, depression and pain, were found to be associated with the high level of cancer-related fatigue trajectory.

Strengths of this study are the nationwide study design, up to 5-year follow-up, validated and repeated measurements of cancer-related fatigue, and comprehensive and robust data collection including not only sociodemographic and clinical factors but also more subjective, patient-reported variables. Limitations of this study should also be acknowledged. First, the risk of selection biases may arise since some eligible patients were not reachable, declined to consent, or were too weak to join. Attrition during the follow-up is another issue when patients dropped out due to death, cancer recurrence, or unknown reasons. In addition, some of the included patients did not have data for all follow-up time points but were still included in the analysis. Some of the missing were due to the surgical calendar period (e.g., patients who underwent surgery in 2019 have not reached the 5-year follow-up), which is an observed variable and may have limited influence on fatigue measurements during the study period. Growth mixture models enable using all available information and can provide robust estimates given the missing data was at random (missing depends on observed variables). Second, cancer-related fatigue level might also be influenced by cancer recurrence and the accompanied treatments during follow-up, which were not available in this cohort. Such patients may have higher level of fatigue, and could contribute to or cluster in the high-level fatigue trajectory. Some lifestyle and biochemical factors that might influence cancer-related fatigue were also not available, and the multivariable analyses were exploratory in nature without targeted confounder adjustment and complex interaction terms. Thus, unmeasured or residual confounding is inevitable in this observational study. A large number of factors including important patient-reported outcomes were incorporated in the analysis and should relieve the concern to some extent. But some of the involved variables might be intercorrelated and the potential implications were still unknown. Third, a matched background population was used to calculate a proxy baseline fatigue measurement for each oesophageal cancer patient because it is not practical to obtain fatigue measurements at baseline before cancer diagnosis. In addition, without measurements before and during the first year after the surgery, no evolution pattern could be modelled during the treatment and the acute recovery period. Besides, though validated questionnaires were used, the measurement of the fatigue level depended on the subjective individual perceptions of this symptom and inherent variability might exist, which could not be captured by the mean score estimate for each trajectory. Furthermore, the longitudinal change of the subjective fatigue perception, i.e., response shift [42], could further complicate the variability that was difficult to account for. Finally, the individual trajectory of each patient is not fixed and patients were assigned to a trajectory with a posterior probability. Such uncertainty may lead to underestimation of the following associations but was partly accounted for by using weighted regression methods.

To our knowledge, this is one of the first and largest studies regarding longitudinal cancer-related fatigue trajectories among oesophageal cancer patients. Two distinct trajectories were identified for overall and dimensional cancer-related fatigue, while for cognitive fatigue and social sequelae, only one trajectory was found. The numbers of the identified trajectories in this study were less than in previous studies on fatigue among breast cancer [43] and Hodgkin’s lymphoma [38] survivors. This may be partly explained by the limit of the minimum sample size (≥15%) required within each trajectory was larger in the current study than in other studies, which guaranteed sounder and more stable estimates. The fatigue level of the identified trajectories was rather persistent or even deteriorating during the follow-up, without a sign of symptom alleviation, especially in the trajectory with a high level of cancer-related fatigue. Such findings suggest the possibility of using an initial measurement for early identification of the patients within long-term poor cancer-related fatigue trajectory. Personalised cancer survivorship care with timely intervention after early patient identification may prevent them from the long-term fatigue burden.

This study found older patients were associated with a low level of cancer-related fatigue trajectories. However, patients of older age are less prone to be affected by emotional distress after cancer diagnosis [44–46], and the association in this study disappeared after adjusting for patient-reported outcome factors, suggesting that the effect of age might be due to confounding or mediation. Proxy fatigue score before cancer diagnosis did not influence long-term cancer-related fatigue in this study, while findings from other studies suggested that fatigue measurements after diagnosis influenced the longitudinal fatigue development [32, 34, 38], which indicated that the timing when measuring the baseline (initial) fatigue level seem to play an important role. Vulnerable patients with more comorbidities [32, 43], advanced tumour stage [47], and postoperative complications [16, 48] are associated with having a higher level of cancer-related fatigue, which was also seen in this study.

Cancer-related fatigue seldom occurs alone and often clusters with other symptoms, including anxiety, depression, pain, and insomnia [31, 34, 43, 49]. This study also found that patients reporting a higher level of these symptoms one year after the surgery were at an increased risk of having a poor long-term cancer-related fatigue trajectory. This calls for the timely monitoring of clustered post-treatment symptoms. Since no Gold-Standard treatment is established for cancer-related fatigue, a comprehensive interventional programme, such as cognitive-behavioural therapy, addressing concurrent symptoms that are easier to modify may help not only relieve the fatigue symptom but also improve the overall cancer survivorship [50]. In addition, given the current healthcare workforce shortage [51, 52], the development and implementation of digital self-management applications are expected to share the workload and are recommended to be incorporated into post-discharge care. The one-time measurement of physical activity did not influence the longitudinal fatigue patterns [33, 43], which underlined the effect of long-term systematic exercise [53].

Understanding the separable long-term development of cancer-related fatigue is the basis for patient stratification and tailored care among cancer survivors. This population-based and longitudinal cohort study revealed distinct 5-year cancer-related fatigue trajectories among oesophageal cancer survivors. More comorbidities, advanced pathological tumour stage, postoperative complications, and self-reported symptoms of anxiety, depression, and pain, seem to influence the fatigue trajectory belonging. Early identification of high-risk patients using the factors found to be associated with a high level of cancer-related fatigue trajectory, and a personalised and comprehensive interventional programme targeting the clustered symptoms are warranted.

### Supplementary information

Supplementary material

STROBE_checklist_cohort

## Data Availability

The datasets generated and analysed in the current study are not publicly available due to ethical restrictions but are available from the corresponding author on reasonable request.

## Code availability

All statistical analyses were calculated using standard methods, therefore the code is not provided.
