# Supplementary material for: Cancer-related fatigue trajectories up to 5 years after curative treatment for oesophageal cancer
Source: Br J Cancer. 2023 Dec 22;130(4):628–37. doi: 10.1038/s41416-023-02551-0 (PMC10876982; doi:10.1038/s41416-023-02551-0)
Supplement: Supplementary file 1 — Supplementary material [file 41416_2023_2551_MOESM1_ESM.pdf]

Title: Cancer-related fatigue trajectories up to 5 years in survivors after oesophageal cancer surgery

Authors:

Zhao Cheng[1], Asif Johar[1], Magnus Nilsson[2,3], Anna Schandl[1], Pernilla Lagergren[1,4*]

Affiliations:

[1] Surgical Care Science, Department of Molecular medicine and Surgery, Karolinska Institutet, Karolinska University Hospital, Stockholm, Sweden

[2] Division of Surgery, Department of Clinical Science, Intervention and Technology (CLINTEC), Karolinska Institutet, Stockholm, Sweden

[3] Department of Upper Abdominal Diseases, Karolinska University Hospital, Stockholm, Sweden

[4] Department of Surgery and Cancer, Imperial College London, London, United Kingdom

***Correspondence to:** Professor Pernilla Lagergren, Department of Molecular medicine and Surgery, Retzius Street 13A, 4th Floor, Karolinska Institutet, 171 77 Stockholm, Sweden (e-mail: pernilla.lagergren@ki.se)

Supplementary Materials - Index

Supplementary Results

Table 1	*pag. 2*
Table 2	*pag. 4*
Table 3	*pag. 6*
Figure 1	*pag. 8*
Table 4	*pag. 9*

Table 1. Characteristics of 409 patients who had at least one measurement of cancer-related fatigue after surgery for oesophageal cancer

	Number	%
**Age**		
Mean (standard deviation)	67.2 (8.3)	-
**Sex**		
Female	34	8.3
Male	375	91.7
**Education level (years)**		
<9	100	24.5
9-12	190	46.5
>12	117	28.6
Missing	2	0.4
**Proxy baseline QLQ-C30 fatigue** [1]		
Mean (standard deviation)	15.2 (7.0)	-
**Charlson comorbidity index**		
0	166	40.6
1	134	32.8
≥2	96	23.5
Missing	13	3.1
**Tumour histology**		
Squamous cell carcinoma	56	13.7
Adenocarcinoma	352	86.1
Missing	1	0.2
**Chemo(radio)therapy**		
No	79	19.3
Yes	329	80.4
Missing	1	0.2
**Pathological tumour stage**		
0-I	137	33.5
II	125	30.6
III-IV	142	34.7
Missing	5	1.2
**Clavien–Dindo classification**		
0–I	148	36.2
II–IIIa	147	35.9
IIIb–IV	102	24.9
Missing	12	2.9
**HADS anxiety** [2]		
No	359	87.8
Yes	44	10.8
Missing	6	1.4
**HADS depression** [2]		
No	355	86.8
Yes	48	11.7
Missing	6	1.5

**QLQ-C30 pain** [1]		
Mean (standard deviation)	19.6 (25.0)	-
Number of missing	5	1.2
**QLQ-C30 insomnia** [1]		
Mean (standard deviation)	23.8 (31.0)	-
Number of missing	5	1.2
**IPAQ physical activity** [3]		
Low	107	26.2
Moderate	123	30.1
High	116	28.4
Missing	63	15.4
**Preoperative BMI adjusted weight loss grading system** [4]		
0	142	34.7
1	48	11.7
2	67	16.4
3	74	18.1
4	35	8.6
Missing	43	10.5

[1] QLQ-C30: Quality of Life Core Questionnaire.
[2] HADS: Hospital Anxiety and Depression Scale.
[3] IPAQ: International Physical Activity Questionnaire.
[4] BMI: body mass index.

Table 2. Fit statistics for model comparison of cancer-related fatigue

	Trajectory number	Latent variance	Latent mean	Residual variance	AIC [1]	BIC [2]	Adjusted BIC [3]	Entropy	P for VLMR [4]	P for aLMR [5]	Note
**QLQ-C30 fatigue [6]**	1	i s	Free	Free	13695.18	13743.34	13705.27	0			
	1	i s q	Free	Free	13688.88	13753.1	13702.33	0			
	1	i s	Free	Same within class	13716.67	13740.76	13721.72	0			
	2	i s	Free	Free	13656.02	13716.23	13668.63	0.691388	0.0522	0.0593	
	**2**	**i s**	**Free**	**Same within class**	**13528.83**	**13568.97**	**13537.23**	**0.586273**	**0.0004**	**0.0006**	
	3	i s	Free	Free	13638.59	13710.84	13653.72	0.682721	0.0399	0.0454	One trajectory with 11.9% sample
	3	i s	Free	Same within class							Model not terminated normally
**QLQ-FA12 overall fatigue [7]**	1	i s	Free	Free	12723	12771.13	12733.05	0			
	1	i s	Free	Same within class	12732.52	12756.59	12737.55	0			
	2	i s	Free	Free	12629.61	12689.78	12642.19	0.77472	0.1216	0.1325	
	**2**	**i s**	**Free**	**Same within class**	**12288.36**	**12328.47**	**12296.74**	**0.76935**	**0**	**0**	
	3	i s@0	Free	Free	12562.36	12626.54	12575.76	0.76281	0.0827	0.0909	One trajectory with 11.7% sample
	3	i s	Free	Same within class	12150.87	12207.02	12162.6	0.73744	0.0175	0.0199	
	4	i s@0	Free	Free	12528.7	12604.91	12544.62	0.784864	0.0702	0.0785	One trajectory with 4.0% sample
	4	i s	Free	Same within class	12073.39	12145.59	12088.48	0.767963	0.0014	0.0017	One trajectory with 10.2% sample
**QLQ-FA12 physical fatigue [7]**	1	i s@0	Free	Free	13561.98	13602.09	13570.36	0			
	1	i s@0	Free	Same within class	13577.71	13593.76	13581.06	0			
	2	i s@0	Free	Free	13503.78	13555.93	13514.68	0.749675	0.0001	0.0001	
	**2**	**i s@0**	**Free**	**Same within class**	**13293.01**	**13325.1**	**13299.71**	**0.654866**	**0**	**0**	
	3	i s@0	Free	Free	13494.36	13558.54	13507.77	0.703115	0.1764	0.1922	One trajectory with 14.2% sample
	3	i s	Free	Same within class	13204.67	13260.83	13216.41	0.734776	0.0013	0.0016	One trajectory with 10.5% sample
**QLQ-FA12 emotional fatigue [7]**	1	i s	Free	Free	13650.82	13698.96	13660.88	0			
	1	i s	Free	Same within class	13648.32	13672.39	13653.35	0			
	2	i s@0	Free	Free	13524.6	13576.74	13535.49	0.820059	0.0688	0.0772	
	**2**	**i s**	**Free**	**Same within class**	**12784.3**	**12824.41**	**12792.68**	**0.888681**	**0.0005**	**0.0006**	
	3	i s@0	Free	Free	13419.01	13483.19	13432.42	0.797529	0.1072	0.1199	

	3	i s	Free	Same within class							Model not terminated normally
**QLQ-FA12 cognitive fatigue [7]**	1	i s	Free	Free	12668.86	12717	12678.92	0			
	**1**	**i s**	**Free**	**Same within class**	**12674.82**	**12698.89**	**12679.85**	**0**			
	2	i s	Free	Free	12445.89	12506.06	12458.47	0.937651	0.0308	0.036	One trajectory with 11.9% sample
	2	i s	Free	Same within class							Model not terminated normally
**QLQ-FA12 interference with daily life**	1	i s@0	Free	Free	14274.16	14314.27	14282.54	0			
	1	i s	Free	Same within class	14272.9	14296.97	14277.93	0			
	2	i@0 s	Free	Free	14212.42	14264.57	14223.31	0.842518	0	0	
	**2**	**i s**	**Free**	**Same within class**	**14013.4**	**14053.51**	**14021.78**	**0.71149**	**0**	**0**	
	3	i s	Free	Same within class	14009.39	14065.54	14021.12	0.790816	0.7732	0.7797	One trajectory with 1% sample
	3	i s	Free	Same within class	14189.94	14254.12	14203.35	0.701441	0.3209	0.3394	One trajectory with 14.8% sample
**QLQ-FA12 social sequelae**	**1**	**i s**	**Free**	**Free**	**13807.93**	**13856.04**	**13817.96**	**0**			
	1	i s	Free	Same within class	13864.95	13889	13869.96	0			
	2	i s	Free	Free	13534.16	13594.29	13546.7	0.999501	0.0001	0.0002	One trajectory with 3% sample
	2	i s	Free	Same within class							Model not terminate normally

[1] AIC: Akaike Information Criterion.
[2] BIC: Bayesian Information Criterion.
[3] Sample-size adjusted BIC.
[4] VLMR: Vuong-Lo-Mendell-Rubin test.
[5] aLMR: adjusted Lo-Mendell-Rubin test.
[6] QLQ-C30: Quality of Life Core Questionnaire.
[7] QLQ-FA12: Quality of Life Fatigue Questionnaire.
Models marked in bold are the selected model.

Table 3. Odds ratios (95% confidence intervals) between sociodemographic, clinical, and patient-reported outcome factors (furtherly included preoperative body mass index (BMI) adjusted weight loss grading system and IPAQ [1] physical activity) in relation to cancer-related fatigue [1] trajectories after surgery for oesophageal cancer

	QLQ-C30 fatigue [2]	QLQ-FA12 overall fatigue [3]	QLQ-FA12 physical fatigue [3]	QLQ-FA12 emotional fatigue [3]	QLQ-FA12 nterference with daily life [3]
**Reference trajectory**	Low	Low	Low	Low	Low
**Outcome trajectory**	High	High	High	High	High
**Age**					
Continuous	1.00 (0.96-1.04)	1.02 (0.98-1.06)	1.01 (0.98-1.05)	1.01 (0.97-1.04)	1.02 (0.98-1.06)
**Sex**					
Female	1.00 (Reference)	1.00 (Reference)	1.00 (Reference)	1.00 (Reference)	1.00 (Reference)
Male	1.30 (0.41-4.14)	1.45 (0.40-5.21)	0.74 (0.24-2.31)	0.54 (0.15-1.95)	0.59 (0.19-1.84)
**Education level (years)**					
<9	1.00 (Reference)	1.00 (Reference)	1.00 (Reference)	1.00 (Reference)	1.00 (Reference)
9-12	0.91 (0.41-2.02)	0.85 (0.36-2.01)	0.68 (0.31-1.52)	1.14 (0.53-2.44)	1.11 (0.47-2.60)
>12	0.65 (0.31-1.35)	0.79 (0.36-1.74)	0.71 (0.35-1.44)	0.92 (0.47-1.80)	0.93 (0.43-2.03)
**Proxy baseline QLQ-C30 fatigue [2]**					
Continuous	0.99 (0.94-1.04)	0.97 (0.92-1.03)	0.99 (0.94-1.04)	0.99 (0.94-1.04)	0.98 (0.93-1.04)
**Charlson comorbidity index**					
0	1.00 (Reference)	1.00 (Reference)	1.00 (Reference)	1.00 (Reference)	1.00 (Reference)
1	0.78 (0.38-1.62)	0.71 (0.32-1.57)	0.66 (0.33-1.36)	0.97 (0.50-1.87)	0.99 (0.46-2.14)
≥2	1.45 (0.57-3.69)	**2.74 (1.02-7.35)**	1.97 (0.79-4.93)	1.37 (0.55-3.39)	**3.22 (1.22-8.48)**
**Tumour histology**					
Squamous cell carcinoma	1.00 (Reference)	1.00 (Reference)	1.00 (Reference)	1.00 (Reference)	1.00 (Reference)
Adenocarcinoma	1.07 (0.45-2.58)	1.22 (0.46-3.20)	1.05 (0.44-2.54)	1.34 (0.55-3.24)	0.85 (0.35-2.07)
**Chemo(radio)therapy**					
No	1.00 (Reference)	1.00 (Reference)	1.00 (Reference)	1.00 (Reference)	1.00 (Reference)
Yes	1.00 (0.47-2.16)	1.15 (0.51-2.63)	1.08 (0.51-2.29)	1.12 (0.54-2.32)	1.24 (0.55-2.82)
**Pathological tumour stage**					
0-I	1.00 (Reference)	1.00 (Reference)	1.00 (Reference)	1.00 (Reference)	1.00 (Reference)
II	1.62 (0.78-3.35)	1.20 (0.54-2.64)	1.10 (0.54-2.22)	1.00 (0.51-1.94)	1.24 (0.58-2.66)
III-IV	**2.03 (1.01-4.09)**	**3.23 (1.55-6.71)**	1.91 (0.97-3.77)	1.08 (0.56-2.07)	1.65 (0.79-3.44)
**Clavien–Dindo classification**					
0–I	1.00 (Reference)	1.00 (Reference)	1.00 (Reference)	1.00 (Reference)	1.00 (Reference)
II–IIIa	1.72 (0.89-3.34)	1.76 (0.88-3.53)	1.29 (0.68-2.44)	**2.55 (1.37-4.73)**	1.31 (0.66-2.59)
IIIb–IV	**2.38 (1.11-5.12)**	1.72 (0.76-3.86)	1.84 (0.89-3.83)	**2.07 (1.03-4.19)**	1.16 (0.52-2.58)
**HADS anxiety [4]**					
No	1.00 (Reference)	1.00 (Reference)	1.00 (Reference)	1.00 (Reference)	1.00 (Reference)
Yes	1.67 (0.57-4.91)	**9.00 (2.28-35.57)**	2.75 (0.88-8.58)	**8.88 (1.10-71.65)**	**3.73 (1.34-10.37)**
**HADS depression [4]**					
No	1.00 (Reference)	1.00 (Reference)	1.00 (Reference)	1.00 (Reference)	1.00 (Reference)
Yes	**6.39 (2.01-20.30)**	**28.18 (4.86-163.25)**	**16.54 (3.38-80.79)**	**15.35 (1.64-143.32)**	**7.28 (2.56-20.72)**
**QLQ-C30 pain [2]**					
Continuous	**1.02 (1.01-1.03)**	**1.03 (1.01-1.04)**	**1.02 (1.01-1.04)**	**1.03 (1.01-1.04)**	1.01 (1.00-1.03)

**QLQ-C30 insomnia [2]**					
Continuous	**1.01 (1.00-1.02)**	1.01 (1.00-1.02)	1.01 (1.00-1.01)	**1.02 (1.00-1.03)**	1.01 (1.00-1.02)
**Preoperative BMI adjusted weight loss grading system**					
0	1.00 (Reference)	1.00 (Reference)	1.00 (Reference)	1.00 (Reference)	1.00 (Reference)
1	1.74 (0.72-4.21)	2.06 (0.79-5.36)	1.50 (0.62-3.63)	1.92 (0.82-4.51)	0.94 (0.35-2.55)
2	1.52 (0.69-3.35)	1.25 (0.53-2.90)	1.09 (0.50-2.41)	1.43 (0.67-3.05)	1.21 (0.54-2.73)
3	0.99 (0.46-2.15)	1.16 (0.52-2.60)	1.18 (0.57-2.47)	0.97 (0.48-1.96)	1.30 (0.59-2.84)
4	0.72 (0.24-2.19)	0.32 (0.09-1.17)	0.56 (0.19-1.66)	**0.33 (0.11-0.97)**	0.37 (0.11-1.30)
**IPAQ physical activity [1]**					
Low	1.00 (Reference)	1.00 (Reference)	1.00 (Reference)	1.00 (Reference)	1.00 (Reference)
Moderate	0.95 (0.46-1.96)	1.43 (0.67-3.06)	0.97 (0.48-1.96)	1.33 (0.68-2.62)	0.98 (0.47-2.07)
High	1.43 (0.69-2.94)	1.21 (0.55-2.66)	1.03 (0.50-2.08)	1.24 (0.62-2.47)	0.98 (0.46-2.08)

[1] IPAQ: International Physical Activity Questionnaire.
[2] QLQ-C30: Quality of Life Core Questionnaire.
[3] QLQ-FA12: Quality of Life Fatigue Questionnaire.
[4] HADS: Hospital Anxiety and Depression Scale.

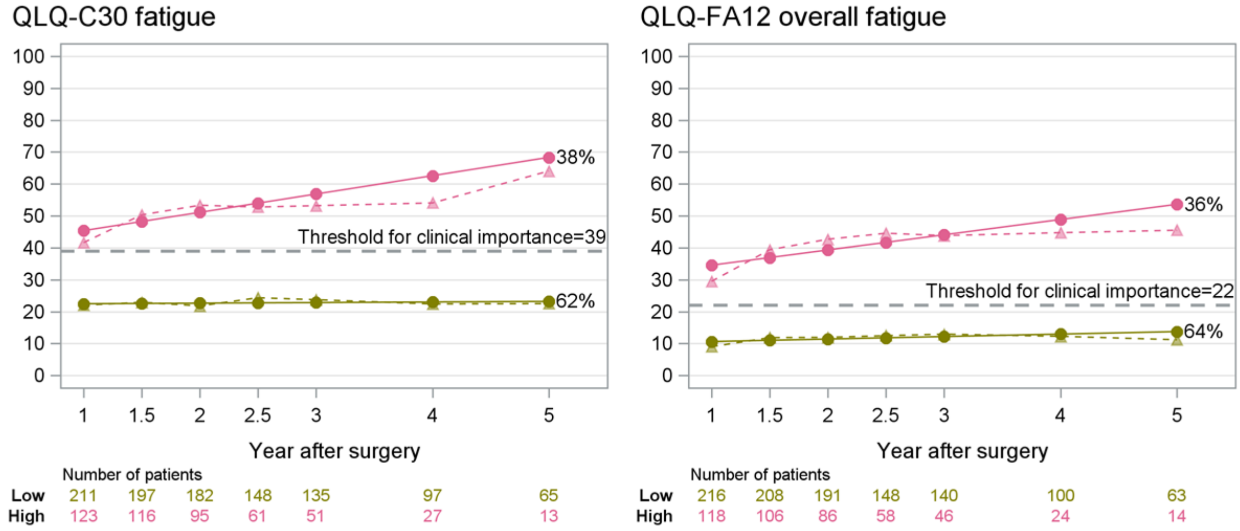

Figure 1. Cancer-related fatigue trajectories after surgery for oesophageal cancer (Sensitivity analyses: excluding patients who had a single measurement for cancer-related fatigue)

1 Solid lines represent estimated means. Dotted lines represent sample means.
2 The percentage after each trajectory is the final patient proportion for the trajectory category based on the most likely trajectory membership.
3 Grey dashed lines represent thresholds for clinical importance (Giesinger JM, et al: J Clin Epidemiol 2020; Friedrich M, et al: Qual Life Res 2019).

Table 4. Odds ratios (95% confidence intervals) between sociodemographic, clinical, and patient-reported outcome factors in relation to cancer-related fatigue trajectories after surgery for oesophageal cancer (Sensitivity analyses: excluding patients who had a single measurement for cancer-related fatigue)

	QLQ-C30 fatigue [1]	QLQ-FA12 overall fatigue [2]
**Reference trajectory**	Low	Low
**Outcome trajectory**	High	High
**Age**		
Continuous	1.00 (0.97-1.04)	1.01 (0.97-1.05)
**Sex**		
Female	1.00 (Reference)	1.00 (Reference)
Male	1.17 (0.41-3.31)	1.75 (0.55-5.58)
**Education level (years)**		
<9	1.00 (Reference)	1.00 (Reference)
9-12	0.67 (0.33-1.34)	1.11 (0.53-2.32)
>12	0.75 (0.35-1.60)	0.89 (0.39-2.02)
**Proxy baseline QLQ-C30 fatigue [1]**		
Continuous	1.01 (0.96-1.06)	1.00 (0.95-1.06)
**Charlson comorbidity index**		
0	1.00 (Reference)	1.00 (Reference)
1	0.96 (0.48-1.94)	0.91 (0.43-1.93)
≥2	1.18 (0.49-2.87)	1.87 (0.74-4.73)
**Tumour histology**		
Squamous cell carcinoma	1.00 (Reference)	1.00 (Reference)
Adenocarcinoma	0.85 (0.37-1.96)	1.40 (0.56-3.49)
**Chemo(radio)therapy**		
No	1.00 (Reference)	1.00 (Reference)
Yes	1.04 (0.50-2.15)	1.83 (0.83-4.06)
**Pathological tumour stage**		
0-I	1.00 (Reference)	1.00 (Reference)
II	1.80 (0.89-3.61)	1.38 (0.65-2.92)
III-IV	**2.84 (1.44-5.61)**	**3.45 (1.71-6.96)**
**Clavien–Dindo classification**		
0–I	1.00 (Reference)	1.00 (Reference)
II–IIIa	1.53 (0.80-2.90)	1.82 (0.93-3.59)
IIIb–IV	1.83 (0.89-3.76)	1.84 (0.86-3.90)
**HADS anxiety [3]**		
No	1.00 (Reference)	1.00 (Reference)
Yes	2.22 (0.71-6.96)	**11.52 (2.59-51.24)**
**HADS depression [3]**		
No	1.00 (Reference)	1.00 (Reference)
Yes	**6.92 (1.94-24.72)**	**19.61 (3.80-101.09)**
**QLQ-C30 pain [1]**		
Continuous	**1.02 (1.01-1.03)**	**1.03 (1.01-1.04)**
**QLQ-C30 insomnia [1]**		

	Continuous	**1.01 (1.00-1.02)**	1.01 (1.00-1.02)

[1] QLQ-C30: Quality of Life Core Questionnaire.
[2] QLQ-FA12: Quality of Life Fatigue Questionnaire.
[3] HADS: Hospital Anxiety and Depression Scale.
Bold results are statistically significant.
